# Using parent feedback: A qualitative study of residents’ and physician-educators’ perspectives

**DOI:** 10.1007/s40037-017-0393-6

**Published:** 2017-12-18

**Authors:** Kaylee Eady, Katherine A. Moreau

**Affiliations:** 1 Children’s Hospital of Eastern Ontario Research Institute, Ottawa, Ontario Canada; 2 Faculty of Education, University of Ottawa, Ottawa, Ontario Canada

**Keywords:** Feedback, Medical education, Parents, Qualitative research

## Abstract

**Introduction:**

Patients and family members can contribute to resident assessment in competency-based medical education. However, few studies have examined the use of patient/family member feedback generated from questionnaire-based assessments. To implement appropriate assessment strategies and optimize feedback use, we need to understand how residents and physician-educators would use feedback from these stakeholders. This study aimed to understand how paediatric residents and physician-educators would use parent feedback generated from questionnaire-based assessments.

**Methods:**

This study was conducted at a paediatric academic health science centre. We held dyadic interviews with six residents and six physician-educators. A three-step approach was used to analyze the data: data reduction, data display, and conclusions/verifications. We developed an initial coding scheme, conducted an in-depth review of the data and coded it, finalized our coding scheme, and identified categories.

**Results:**

Participants described that they would use parent feedback to: (a) provide additional direct observations of residents’ performances, (b) teach and coach residents, (c) assess residents’ overall performance and progression, and (d) encourage resident self-assessment and behaviour change.

**Discussion:**

Parents directly observe residents as they interact with them and their children and, therefore, can provide feedback on residents’ performances. Residency programs should include parent feedback and promote and facilitate its use by residents and physician-educators.

**Conclusion:**

This study provides an initial understanding of how paediatric residents and physician-educators would use parent feedback if they were to receive it. This information, combined with future research, can inform the development and implementation of parent feedback strategies in competency-based medical education.

## What this paper adds

Patients and their family members can make significant contributions to resident assessment, especially within competency-based medical education. However, few studies have examined residents’ and physician-educators’ use of feedback from these key stakeholders. It is important to understand feedback use in order to implement appropriate patient and family member assessment strategies that optimize use. This study provides insight into how paediatric residents and physician-educators would use parent feedback on residents’ skills and abilities generated from questionnaire-based assessments, ultimately informing their clinical practices and professional development.

## Introduction

Patients and family members can meaningfully assess residents’ professionalism, collaboration, interpersonal abilities, and communication skills [1, 2]. Their involvement in resident assessment is important given the prevalence of patient- and family-centred care in health contexts as well as its impact on healthcare quality and health outcomes [3]. The Institute for Patient- and Family-Centered Care stresses that all aspects of professional practice should incorporate patient- and family-centred care, including medical education [4]. Educators and researchers further emphasize these stakeholders’ roles in medical education and the need for leaners to learn with and from them [5, 6]. Efforts are underway to involve patients and family members in learner assessment [7], including, for example, the assessment of learners’ communication skills via questionnaires [8–11]. However, research is needed to understand how residents and physician-educators would use the feedback generated from questionnaires to improve clinical practices.

As educators and researchers work to involve patients and family members in medical education, others are working to transition from time-based to competency-based medical education (CBME) [12]. Of interest to this study, paediatrics residency programs in Canada are currently in the midst of transitioning to CBME [13] and therefore, educators and researchers are looking for new and innovative assessment strategies for residents [14]. To effectively improve residents’ skills, direct observation of performance is the most accurate assessment method and allows for timely feedback [14, 15]. Performance-based assessment, especially through direct observation in clinical settings, is essential but not well studied amidst the above-mentioned curricular changes [16, 17].

For assessment to be effective, residents must utilize the feedback generated from it. Many factors can influence the use of feedback, such as its timing, understanding what to do with it, and one’s perceived need for and openness to receiving it [18–20]. Residents’ perceptions of the assessors’ credibility and the data collected are central to their use of feedback [21–23]. It is therefore important to understand how residents and physician-educators would incorporate it into their practices. This understanding would facilitate the implementation of appropriate assessment strategies within paediatric settings that adhere to or are transitioning to CBME and optimize feedback use. Within paediatrics, medical educators also need to be mindful of the important role that patients’ parents (i. e., caregivers, guardians) can play in resident assessment and understand how residents and physician-educators would use parent feedback.

Parents are experts on residents’ interactions with them and their children, as they directly witness residents’ performance. Therefore, parents are considered important sources of feedback in multisource feedback [14, 24], an assessment method that includes the perspectives and observations of various assessors [25, 26]. That said, a review of assessment data found that only 34% of Accreditation Council for Graduate Medical Education specialties and subspecialty programs in the United States (US) included family members as assessors, but that this involvement was increasingly prevalent in primary care specialties, including paediatrics, where learners had multiple interactions with family members [27]. In the Canadian paediatric context, parents typically provide feedback informally when physician-educators request it. This informal parent feedback is a potential reflection of the limited research on parent involvement in assessment processes and on the development of educational assessment tools to facilitate this involvement [28]. Few studies have examined residents’ and physician-educators’ use of parent feedback. As such, the aim of this study was to answer: How would paediatric residents and physician-educators use parent feedback generated from questionnaire-based assessments?

## Methods

To fulfil the study aim, we used a basic interpretive qualitative approach [29], within a constructivist viewpoint. This approach seeks to understand a phenomenon from the perspective of those who experience it and provides a descriptive account of the findings. As our understanding of the potential use of parent feedback in this context is limited, this approach allowed us to describe this use from the perspective of paediatric residents and physician-educators. However, we recognize that our backgrounds as PhD-trained health professions education researchers and advocates for patient and family engagement in educational processes are also reflected throughout this study and our interpretation of the findings. We obtained approval from the study site’s Research Ethics Board prior to study commencement.

### Setting and participants

We conducted this study at a standalone, urban paediatric academic health science centre in Ontario, Canada. This centre is committed to patient- and family-centred care. The paediatrics residency program at this centre typically consists of 40 residents and is currently preparing to transition to CBME.

We used purposeful sampling [30] to recruit residents enrolled in the paediatrics residency program as well as physician-educators with resident supervision and assessment responsibilities. We sought to identify individuals with similar relationships to and experiences with resident training and assessment in order to pair them for participation in dyadic interviews. Specifically, we recruited and paired paediatric residents by postgraduate year and physician-educators by number of years of experience supervising and assessing residents in order create a safe interview space for participants to express their opinions and minimize any potential hierarchy issues within each dyad [31]. The research assistant emailed the information letter to paediatric residents on rotation and to physician-educators at the centre via their designated listservs. With this information letter, we invited interested residents and physician-educators to contact the research assistant for additional information and to schedule an interview.

### Data collection

We [KE and KAM] individually conducted the dyadic interviews from October to November 2015 while the research assistant took notes. Dyadic interviews require two interviewees (i. e., two residents or two physician-educators) to interact with one another, engage in dialogue on the topic, and share ideas while answering open-ended questions posed by a moderator [32, 33]. This data collection method allows participants to share their experiences and thoughts on a topic in an in-depth manner that is not always feasible in focus groups, while maintaining and encouraging the active interaction between participants, which is lost in one-on-one interviews.

We used the published assessment literature to inform the development of the interview guide (see Tab. 1), which we piloted with three residents and three physician-educators who did not participate in this study. Written, informed consent was obtained prior to participation. During the interviews, we used in-vivo member checking to verify our understanding of the participants’ discussion and responses. The dyadic interviews lasted 60 min, took place in a private room at the centre, were audio-recorded, and transcribed verbatim by a professional transcriptionist. Following transcription, we verified transcript accuracy by comparing the transcripts with the audio-recordings. We also shared the individual transcripts with the respective participants for their review and feedback, all of whom were accepting of them and did not provide any additional comments [34].

**Table 1:** Dyadic interview questions and example prompts

Participant group	Interview questions
Physician-educators	1. Tell us a little bit about yourselves
	2. If you were to receive feedback from parents/caregivers through a questionnaire for a resident you were supervising, how would you use that feedback?
	*Example prompt: How would it inform what you do as an educator*?
	3. In your opinion, how would the resident use that feedback?
	*Example prompt: How would it inform their training or practices*?
	4. Is there anything else on this topic that you would like to talk about?
Paediatric residents	1. Tell us a little bit about yourselves
	2. If you were to receive feedback from parents/caregivers through a questionnaire, how would you use that feedback?
	*Example prompt: How would it inform your training*?
	3. In your opinion, how would your supervising physician use that feedback?
	*Example prompt: How would it inform what they do as an educator*?
	4. Is there anything else on this topic that you would like to talk about?

### Data analysis

The transcribed interviews were entered into NVivo 11 to organize and manage the data [35]. We independently analyzed and coded the data, ensuring rigor and trustworthiness. A systematic, three-step approach to data analysis was used: data reduction, data display, and conclusions and verifications [36]. Our research question, the assessment literature, and our interview notes and summaries guided our analysis. First we reduced the data by developing an initial coding scheme. Next, we conducted an in-depth review of the transcripts and coded the data as per our initial coding scheme, while allowing new codes to emerge from the data. Then we met to compare and discuss our analysis and made minor revisions to our initial coding scheme. This allowed us to identify categories [37] across the participant groups and ensure that we fulfilled our study purpose. Using the final coding scheme, we created a matrix query in NVivo to display and review the data across the participant groups, allowing us to better understand the findings and draw conclusions. We also extracted supporting quotations for reporting purposes. We created an audit trail to document all coding decisions [34].

## Results

Six paediatric residents and six physician-educators participated in this study. Half of the residents (*n* = 3) were female and two were from each year 1, 2, and 3 of residency. Four of the physician-educators were female. All had experience supervising and assessing residents: two had 1–5 years; two had 6–10 years; and two had 16–20 years of experience.

Paediatric residents and physician-educators believed that they would use parent feedback to: (a) provide additional direct observations of residents’ performances, (b) teach and coach residents, (c) assess residents’ overall performance and progression, and (d) encourage resident self-assessment and behaviour change.

### To provide additional direct observations of residents’ performances

Participants expressed that parent feedback could contribute to resident assessment by providing additional direct observations of their performances. Because physician-educators are not always able to observe residents directly, the physician-educators explained that they sometimes have to make assumptions about residents’ interactions with parents:

*We are making a bit of a leap to say we understand how they interact with us and we have, you know, good vibes versus a little bit of fear. … we perceive that’s how they would carry themselves with the families.*

The residents echoed this idea and added:

*… they [staff] don’t often see you communicate with patients. And if they do, it’s kind of a nerve-wracking experience as well. It’s a very artificial one. … your interaction is completely different from how you would normally do it, mostly because you’re watching your ps and qs cause you’re trying to be so professional for your staff, so that may not be the way you actually are and I think it’s really good to get that feedback … this is actually how I’m going to interact with a patient when they come in.*

### To teach and coach residents

Participants conveyed that parent feedback could provide physician-educators an opportunity to teach and coach residents. They explained that parent feedback would allow physician-educators to review with the residents what went well during their interactions with patients/parents, and how they would use this feedback for ‘timely teaching’. The residents explained:

*… if there was an episode where I was flagrantly unprofessional, flagrantly a bad communicator… I still want to know about that. … if it [assessment] goes to the staff… and [they] go ‘well there was one episode where we don’t think the communication went as well as you could’ve, here are some things that you can work on’ …*

The physician-educators also explained:

*I think that [parent assessments] would provide very valuable feedback that could be read back in a summative fashion to the resident … at the end of the shift to say ‘Okay, so this is the feedback of the parent of Johnny. They’ve identified these two things that you could have done better. Let’s talk about that.’ And it becomes an opportunity. A really teachable moment, right?*

The residents believed that parent feedback could also provide documented examples of areas where residents need improvement:

*So in those instances where there are people who have been identified as potentially unprofessional, but it’s been a difficult way to substantiate that or give any evidence of it and that would be helpful hearing … Like from a parent … ‘Actually that encounter was completely unprofessional and this is why.’*

Likewise, the physician-educators conveyed how parent feedback could prompt them to work more closely with residents on certain skills:

*… if there’s a trend over time of parents saying similar comments or the same kind of ratings … that could be a flag to you … that this is someone that you need to go into the room with them and work together to figure out why the parents are getting that.*

### To assess residents’ overall performance and progression

Participants believed that collecting feedback from a variety of parents over the course of a rotation would be valuable to provide a sense of residents’ overall performance at the end of their rotations, as they advance in their training and develop their competencies. A resident expressed, ‘*I feel like, for me anyway, it would be more useful over the whole block, to see, okay, what were the general themes*.’ Another described a potential delay in receiving supervisor feedback, and how parent feedback, immediately following their rotations, could help them improve their performance while they wait:

*I did one of my rotations in July, I could have been terrible, I don’t know. I’m gonna do two other senior blocks before I get that feedback for the first one. So, I could have really used that information to change how I’m interacting for the next two [rotations] …*

The residents also believed that parent feedback might be helpful to physician-educators as they prepare residents’ final evaluations:

*If our staff have it, and they’re doing our final evals, and they’re kind of helping [with] that to put in specific comments … and … rate us on how we did on our communicator role, like on the final ITER [In-Training Evaluation Report], I can see that being possibly useful to them.*

The physician-educators voiced that ongoing collection of parent feedback would contribute to their understanding of residents’ progression through their rotations. A physician-educator expressed, *‘… have they really changed over time or are they the same as when they came out of medical school when they made the residency?’ *Beyond individual rotations, they also alluded to the idea that parent feedback could contribute to residents’ development throughout their residency. A physician-educator summarized this:

*… hopefully that, even though it’s only a month that we’ve imparted some [knowledge] and made some refinement to some of those softer skills about those CanMEDS roles around collaborator and around communicator and around health advocate … getting evaluations from parents and getting them back to them [residents] … [if it] was actually resulting in a better doctor at the end of the month if not at some point near the end of the residency …*

### To encourage resident self-assessment and behaviour change

Participants believed that parent feedback could inform their self-assessments, subsequently informing their behaviours and practices. They explained how as residents progress through their training, they make their own training decisions. A resident spoke to how parent feedback could contribute to this process:

*… you get all these suggestions from the staff about ‘this is how I do it … so therefore, this is how you should do it’. But you have 10 different people who do it 10 different ways, so you kind of pick what you think is best. But it would be really nice from the … receiver, the caregiver or the child who is getting … what we’re putting out for them, how they actually took it. Because I think that is actually the more important thing.*

Another resident described the potential thought process of using parent feedback to inform their learning:

*Negative feedback is, thankfully, it’s more uncommon, but when you do get it, there’s a lot more thinking about negative feedback. And, you know, the process is usually first of all, do I consciously agree with that parent assessment of, or that negative feedback that I’m getting from them … and if I do, well then we say, okay, well what led to that?*

The physician-educators explained that providing residents with parent feedback would provide them with: ‘*… feedback that’s truly of value to them. That they’re going to appreciate, that they’re going to incorporate and use and not feel like it’s just one more thing.’ *Reflecting from a resident perspective, a physician-educator further explained that* ‘… it would be invaluable to know how people perceive you and how people perceive their interaction with you. And what you could work on to do better and what you do well. And you could carry that forward.*’

## Discussion

This study sought to understand how paediatric residents and physician-educators would use parent feedback generated from questionnaire-based assessments. Assessment in CBME demands direct observations of authentic resident-patient-parent interactions. This performance-based assessment is critical for residents’ professional growth to ensure that they can enact the skills they have learned in clinical settings [14, 15, 19, 38]. Moreover, this type of assessment should be done regularly, rather than on a one-off basis, to properly reflect residents’ skills and abilities [16]. We believe parents can provide timely feedback that can be regularly collected via a questionnaire following their interactions with a resident. The immediate use of parent feedback would benefit residents by facilitating appropriate resident recall and opportunity for immediate behaviour change [19]. However, to enable parents to provide feedback, we need to develop and use questionnaires designed specifically for parents in paediatric contexts [28].

Participants’ descriptions of how they would use parent feedback suggest that they view parents as credible sources of feedback. This aligns with research from the US showing that the inclusion of family members as assessors is more prevalent in primary care specialties [27]. The participants noted that parents directly observe residents as they interact with them and their children, and therefore are directly familiar with residents’ performance. These views are important to consider as the credibility of the assessor is a central factor in the use of feedback [21–23]. Further, the participants thought that parent feedback would be important and meaningful for residents’ professional development. These perceptions reflect those of previous research that described learners’ perceptions of service user involvement in health professions education as a positive and rewarding professional development experience [39].

In multisource feedback, an assessment method promoted within CBME [25], family members are viewed as a source of feedback for residents in addition to that from physician-educators, peers, and other health professionals [14, 24, 26]. Research has shown that feedback generated through this questionnaire-based method is generally accepted by learners and that they use it to reflect on and/or make changes to their practices [26]. The participants expressed desire for parent feedback in addition to that from physician-educators. That being said, in the Canadian paediatric context, feedback from family members is typically provided informally and on an ad hoc basis, most often if physician-educators ask for their feedback on residents who interacted with them. In fact, while multisource feedback is used within the paediatrics program in this study, parent feedback is not included in it.

The findings from this study suggest that there is value in incorporating parent feedback into assessment processes across all paediatric residency programs. This would contribute to well-rounded assessments and ensure consistency across programs [40, 41]. Incorporating parent feedback would promote and facilitate the consistent use of this feedback in training environments, all of which are necessary factors in using feedback for self-assessment [22]. Self-assessment plays an integral role in medical education; reflective practice is encouraged and modelled so that residents involve themselves in their own learning and assessment. The literature also highlights that self-assessment should not be done in isolation, but informed by multiple sources of evidence [14, 42]. The participants expressed that they would use parent feedback to inform resident self-assessment and in turn, inform their reflections on their behaviours and practices. Therefore, parent feedback can be a valuable contribution to this process.

This study presents certain limitations that should be considered. It included a limited group of paediatric residents from a single paediatric residency program and a limited group of physician-educators from a single paediatric academic health science centre. Further, this study described residents’ and physician-educators’ perceptions of how they would use parent feedback, and does not report descriptions of their actual use of this feedback.

## Conclusion

Parents can play an important role in paediatric resident assessment. This study provides an initial understanding of how paediatric residents and physician-educators would use parent feedback. This information, combined with future research, can inform the development and implementation of parent feedback strategies. Importantly, it can help promote the use of parent feedback for paediatric resident assessment in CBME.

## References

[1] Abadel FT, Hattab AS (2014). Patients’ assessment of professionalism and communication skills of medical graduates. Bmc Med Educ.

[2] Moreau KA, Eady K, Frank JR (2016). A qualitative exploration of which resident skills parents in pediatric emergency departments can assess. Med Teach.

[3] Committee on Hospital Care, Institute for Patient- and Family-Centered Care (2012). Patient- and family-centered care and the pediatrician’s role. Pediatrics.

[4] Institute for Patient- and Family-Centered Care (2016). Patient- and family-centered care.

[5] Bleakley A, Bligh J (2008). Students learning from patients: let’s get real in medical education. Adv Health Sci Educ Theory Pract.

[6] Towle A, Farrell C, Gaines ME (2016). The patient’s voice in health and social care professional education. Int J Health Gov.

[7] Towle A, Godolphin W (2016). Patient involvement in health professional education: a bibliography 1975—November 2016.

[8] Crossley J, Eiser C, Davies HA (2005). Children and their parents assessing the doctor-patient interaction: a rating system for doctors’ communication skills. Med Educ.

[9] McGraw M, Fellows S, Long A (2012). Feedback on doctors’ performance from parents and carers of children: a national pilot study. Arch Dis Child.

[10] Campbell C, Lockyer J, Laidlaw T, MacLeod H (2007). Assessment of a matched-pair instrument to examine doctor-patient communication skills in practising doctors. Med Educ.

[11] Makoul G, Krupat E, Chang CH (2007). Measuring patient views of physician communication skills: development and testing of the communication assessment tool. Patient Educ Couns.

[12] Frank JR, Snell LS, Cate OT (2010). Competency-based medical education: theory to practice. Med Teach.

[13] Royal College of Physicians and Surgeons of Canada (2017). CBD implementation 2017.

[14] Holmboe ES, Sherbino J, Long DM, Swing SR, Frank JR (2010). The role of assessment in competency-based medical education. Med Teach.

[15] Holmboe ES, Holmboe ES, Hawkins RE (2008). Direct observation by faculty. Practical guide to the evaluation of clinical competence.

[16] Norcini JJ (2005). Current perspectives in assessment: the assessment of performance at work. Med Educ.

[17] Norcini JJ (2017). The role of assessment in supporting the movement toward patient-centred care. Perspect Med Educ.

[18] Jonsson A (2012). Facilitating productive use of feedback in higher education. Act. Learn. High. Educ..

[19] Ramani S, Krackov SK (2012). Twelve tips for giving feedback effectively in the clinical environment. Med Teach.

[20] van de Ridder JM, McGaghie WC, Stokking KM, ten Cate OT (2015). Variables that affect the process and outcome of feedback, relevant for medical training: a meta-review. Med Educ.

[21] Ferguson J, Wakeling J, Bowie P (2014). Factors influencing the effectiveness of multisource feedback in improving the professional practice of medical doctors: a systematic review. BMC Med Educ.

[22] Sargeant J, Armson H, Chesluk B (2010). The processes and dimensions of informed self-assessment: a conceptual model. Acad Med.

[23] Watling C (2014). Cognition, culture, and credibility: deconstructing feedback in medical education. Perspect Med Educ.

[24] Holmboe ES, Ross K (2012). Commentary: realizing the formative potential of multisource feedback in regulatory-based assessment programs. Acad Med.

[25] Lockyer J, Clyman SG, Holmboe ES, Hawkins RE (2008). Multisource feedback (360-degree evaluation). Practical guide to the evaluation of clinical competence.

[26] Lockyer J (2003). Multisource feedback in the assessment of physician competencies. J Contin Educ Health Prof.

[27] Holt KD, Miller RS, Nasca TJ (2010). Residency programs’ evaluations of the competencies: data provided to the ACGME about types of assessments used by programs. J Grad Med Educ.

[28] Moreau KA, Pound CM, Eady K (2015). Pediatric caregiver involvement in the assessment of physicians. BMC Med Educ.

[29] Merriam SB (2002). Qualitative research in practice.

[30] Patton MQ (2015). Qualitative evaluation and research methods.

[31] Morgan DL, Ataie J, Carder P, Hoffman K (2013). Introducing dyadic interviews as a method for collecting qualitative data. Qual Health Res.

[32] Morgan DL, Eliot S, Lowe RA, Gorman P (2016). Dyadic interviews as a tool for qualitative evaluation. Am J Eval.

[33] Sohier R (1995). The dyadic interview as a tool for nursing research. Appl Nurs Res.

[34] Lincoln Y, Guba E (1985). Naturalistic inquiry.

[35] NVivo 11 for Windows. 11 ed. Burlington, MA: QSR International; 2015.

[36] Miles MB, Huberman AM (1994). Qualitative data analysis: an expanded sourcebook.

[37] Morse JM (2015). Analytic strategies and sample size. Qual Health Res.

[38] Ramani S, Post SE, Konings K, Mann K, Katz JT, van der Vleuten C (2017). It’s not just the culture: a qualitative study exploring residents’ perceptions of the impact of institutional culture on feedback. Teach Learn Med.

[39] Morgan A, Jones D (2009). Perceptions of service user and carer involvement in healthcare education and impact on students’ knowledge and practice: a literature review. Med Teach.

[40] Chou S, Lockyer J, Cole G, McLaughlin K (2009). Assessing postgraduate trainees in Canada: are we achieving diversity in methods?. Med Teach.

[41] Lockyer J, Carraccio C, Chan MK (2017). Core principles of assessment in competency-based medical education. Med Teach.

[42] Keister DM, Hansen SE, Dostal J (2017). Teaching resident self-assessment through triangulation of faculty and patient feedback. Teach Learn Med.

